# The genome sequence of the northern brown argus, *Aricia artaxerxes* (Fabricius, 1793)

**DOI:** 10.12688/wellcomeopenres.18664.1

**Published:** 2022-12-23

**Authors:** Sam Ebdon, Konrad Lohse, Wellcome Sanger Institute Tree of Life programme, Wellcome Sanger Institute Scientific Operations: DNA Pipelines collective, Tree of Life Core Informatics collective, Alexandra Jansen Van Rensburg, Darwin Tree of Life Consortium

**Affiliations:** 1 Institute of Ecology and Evolution, University of Edinburgh, Edinburgh, UK; 2 Centre for Biodiversity and Environment Research, Department of Genetics, Evolution and Environment, University College London, London, UK

**Keywords:** Aricia artaxerxes, northern brown argus, genome sequence, chromosomal, Lepidoptera

## Abstract

We present a genome assembly from an individual *Aricia artaxerxes* (the northern brown argus; Arthropoda; Insecta; Lepidoptera; Lycaenidae). The genome sequence is 458 megabases in span. Most of the assembly (99.99%) is scaffolded into 23 chromosomal pseudomolecules, including the assembled Z sex chromosome. The mitochondrial genome has also been assembled and is 15.8 kilobases in length. Gene annotation of this assembly on Ensembl has identified 12,688 protein coding genes.

## Species taxonomy

Eukaryota; Metazoa; Ecdysozoa; Arthropoda; Hexapoda; Insecta; Pterygota; Neoptera; Endopterygota; Lepidoptera; Glossata; Ditrysia; Papilionoidea; Lycaenidae; Polyommatinae; *Aricia*; *Aricia artaxerxes* (Fabricius, 1793) (NCBI txid:91738).

## Background

The northern brown argus or mountain argus, *Aricia artaxerxes* (Fabricius, 1793), is a small Lycaenid butterfly found throughout the Palearctic apart from North Africa and the Iberian Peninsula ( Sañudo-Restrepo *et al.*, 2013). This species is a habitat specialist that is often observed flying on alkaline slopes and grassland amongst the preferred larval host plant, the common rock-rose ( *Helianthemum nummularium*). The northern brown argus is univoltine ( Tolman & Lewington, 1997) and hibernates as a larva. *Aricia artaxerxes* exhibits high intraspecific morphological variation across its range, which has led to taxonomic over-splitting ( Sañudo-Restrepo *et al.*, 2013). However, two genetically and morphologically distinct subspecies are found in the British Isles ( Aagaard *et al.*, 2002): subspecies *artaxerxes* is found in Scotland where the species was first described and is easily distinguishable from the closely related *Aricia agestis* (brown argus) by a white spot on the upper forewing. Subspecies *salmacis (*Durham argus) occurs in northern England and lacks the distinctive white wing spot. The range of *A. artaxerxes salmacis* overlaps with the bivoltine *A. agestis* in northern England, and the two species hybridise in populations where their flight times overlap ( Mallet *et al.*, 2011).

Although *A. artaxerxes* is listed as a species of Least Concern on the IUCN Red List (Europe) ( van Swaay *et al.*, 2010), it is classed as vulnerable on the GB Red List ( Fox *et al.*, 2022) and is locally rare in the British Isles, exhibiting large decreases in both occurrence and abundance over the last 30 years ( Fox *et al.*, 2015). Declines in Britain can be attributed primarily to the loss and/or fragmentation of suitable grassland habitat due to intensified agricultural practices ( Franco *et al.*, 2006). Declines have also been attributed to climate change, as *A. artaxerxes* is shifting its range northwards and upwards in elevation in response to increased temperatures ( Franco *et al.*, 2006). At the same time, there is an increasing risk of hybridisation and/or replacement at its southern range margin, as *A. agestis* expands its range northwards in response to climate change. Finally, phenological shifts in response to climate change in univoltine specialists tend to come at a cost, resulting in population declines and retracting distributions as seen in *A. artaxerxes* ( Macgregor *et al.*, 2019).

*Aricia artaxerxes* has 23 chromosome pairs ( Federley, 1938). The genome of spp. *A. artaxerxes* will be of use to researchers investigating how this habitat specialist responds to climate change.

## Genome sequence report

**Figure 1.**
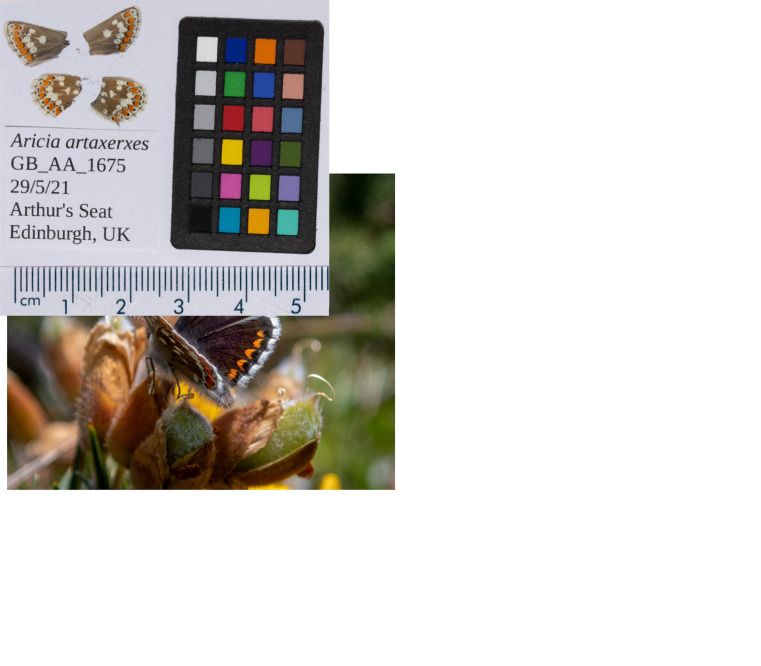
Photographs of GB_AA_1675 (ilAriArta2) used to generate Pacific BioSciences and Hi-C data. **a**) The specimen photographed in the field by Sam Ebdon. **b**) Forewings and hindwings of the *A. artaxerxes* specimen. Dorsal (left) and ventral (right) surface views of wings from the specimen.

The genome was sequenced from an individual male *A. artaxerxes* ( Figure 1) collected from Arthur's Seat, Edinburgh, Scotland (latitude 55.94, longitude –3.16), the type locality of this species. A total of 28-fold coverage in Pacific Biosciences single-molecule HiFi long reads was generated. Primary assembly contigs were scaffolded with chromosome conformation Hi-C data. Manual assembly curation corrected 6 missing/misjoins and removed 3 haplotypic duplications, reducing the assembly length by 0.15% and the scaffold number by 17.24%, and increasing the scaffold N50 by 3.26%.

**Figure 2.**
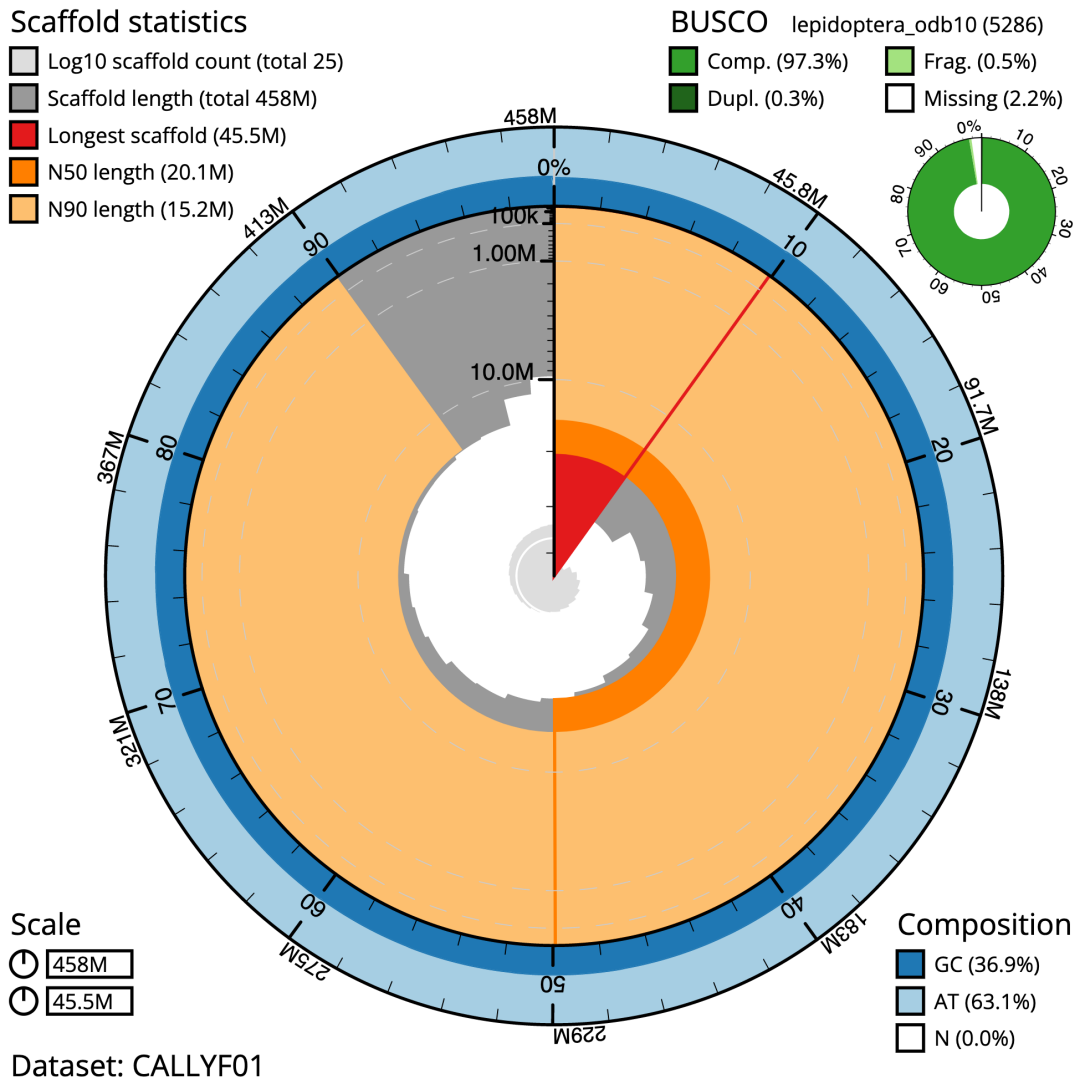
Genome assembly of *Aricia artaxerxes*, ilAriArta2.1: metrics. The BlobToolKit Snailplot shows N50 metrics and BUSCO gene completeness. The main plot is divided into 1,000 size-ordered bins around the circumference with each bin representing 0.1% of the 458,496,882 bp assembly. The distribution of chromosome lengths is shown in dark grey with the plot radius scaled to the longest chromosome present in the assembly (45,450,413 bp, shown in red). Orange and pale-orange arcs show the N50 and N90 chromosome lengths (20,114,245 and 15,169,435 bp), respectively. The pale grey spiral shows the cumulative chromosome count on a log scale with white scale lines showing successive orders of magnitude. The blue and pale-blue area around the outside of the plot shows the distribution of GC, AT and N percentages in the same bins as the inner plot. A summary of complete, fragmented, duplicated and missing BUSCO genes in the lepidoptera_odb10 set is shown in the top right. An interactive version of this figure is available at https://blobtoolkit.genomehubs.org/view/ilAriArta2.1/dataset/CALLYF01/snail.

The final assembly has a total length of 458 Mb in 24 sequence scaffolds with a scaffold N50 of 20 Mb ( Table 1). Most (99.99%) of the assembly sequence was assigned to 23 chromosomal-level scaffolds, representing 22 autosomes (numbered by sequence length) and the Z sex chromosome ( Figure 2– Figure 5; Table 2). The assembly has a BUSCO v5.3.2 ( Manni *et al.*, 2021) completeness of 97.3% (single 96.9% and duplicated 0.3%) using the lepidoptera_odb10 reference set.

**Table 1.** Genome data for *Aricia artaxerxes*, ilAriArta2.1.

Project accession data	
Assembly identifier	ilAriArta2.1
Species	*Aricia artaxerxes*
Specimen	ilAriArta2
NCBI taxonomy ID	91738
BioProject	PRJEB42114
BioSample ID	SAMEA9700886
Isolate information	Male, whole body tissue
Raw data accessions	
PacificBiosciences SEQUEL II	ERR9439488
Hi-C Illumina	ERR9434966
Genome assembly	
Assembly accession	GCA_937612035.1
*Accession of alternate * *haplotype*	GCA_937610355.1
Span (Mb)	458
Number of contigs	53
Contig N50 length (Mb)	14.8
Number of scaffolds	24
Scaffold N50 length (Mb)	20.1
Longest scaffold (Mb)	45.5
BUSCO *	C:97.3%[S:96.9%,D:0.3%], F:0.5%,M:2.2%,n:5286
Genome annotation	
Number of protein-coding genes	12,688
Average length of coding sequence (bp)	16,060.88
Average number of exons per transcript	7.35
Average intron size (bp)	2020.94

* BUSCO scores based on the lepidoptera_odb10 BUSCO set using v5.3.2. C = complete [S = single copy, D = duplicated], F = fragmented, M = missing, n = number of orthologues in comparison. A full set of BUSCO scores is available at https://blobtoolkit.genomehubs.org/view/ilAriArta2.1/dataset/CALLYF01/busco.

**Figure 3.**
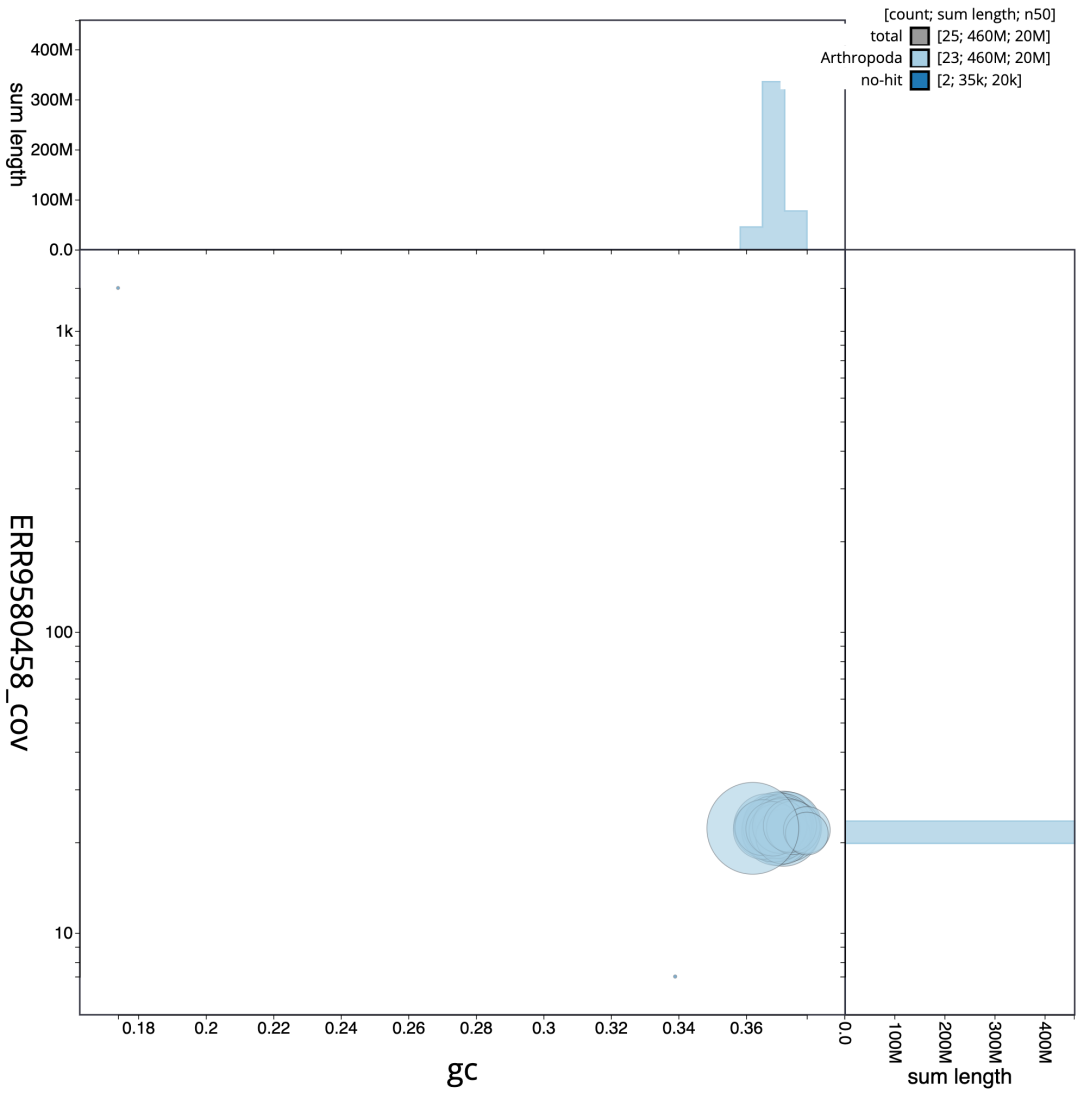
Genome assembly of *Aricia artaxerxes*, ilAriArta2.1: GC coverage. BlobToolKit GC-coverage plot. Chromosomes are coloured by phylum. Circles are sized in proportion to chromosome length. Histograms show the distribution of chromosome length sum along each axis. An interactive version of this figure is available at https://blobtoolkit.genomehubs.org/view/ilAriArta2.1/dataset/CALLYF01/blob.

**Figure 4.**
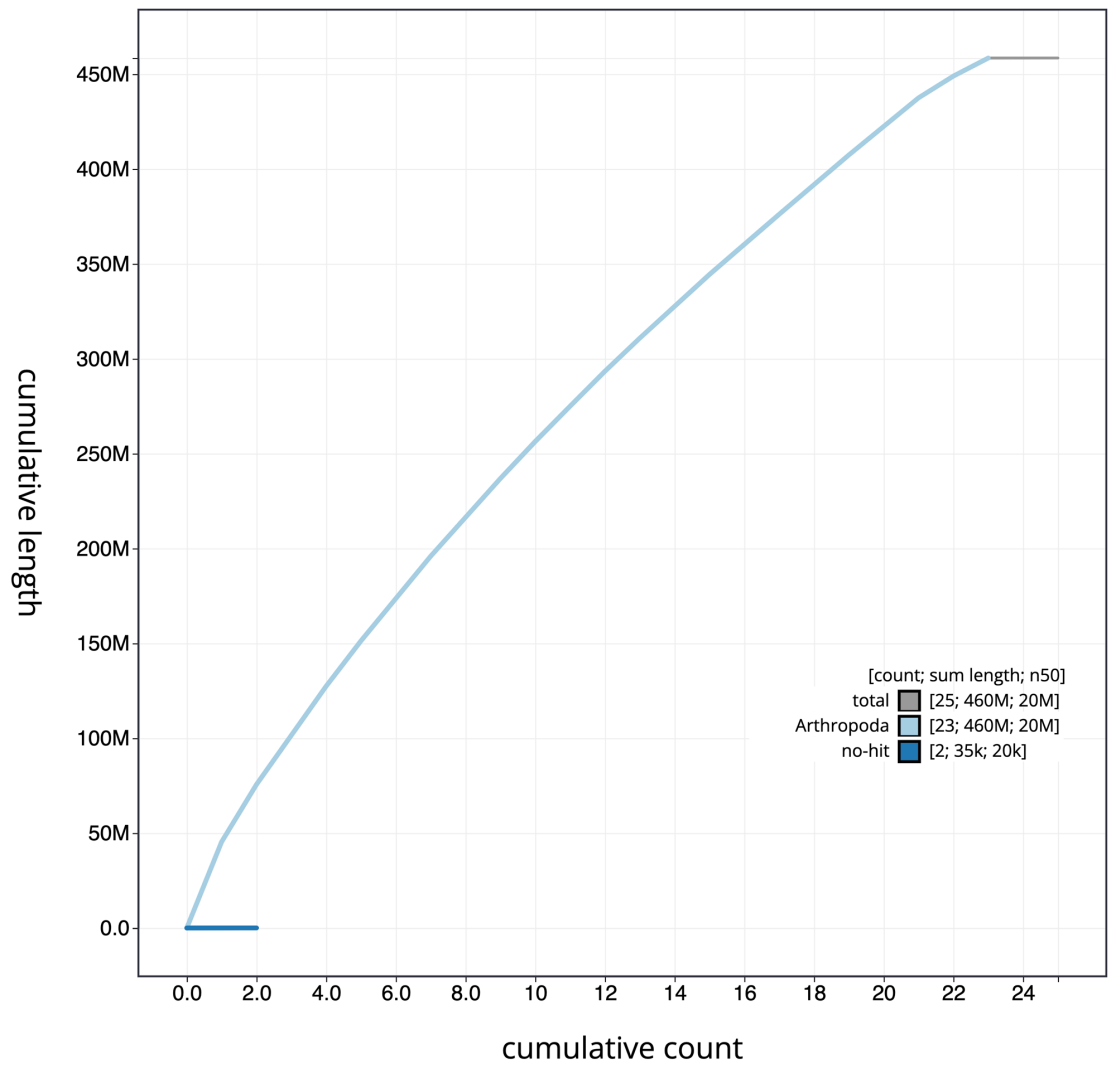
Genome assembly of *Aricia artaxerxes*, ilAriArta2.1: cumulative sequence. BlobToolKit cumulative sequence plot. The grey line shows cumulative length for all chromosomes. Coloured lines show cumulative lengths of chromosomes assigned to each phylum using the buscogenes taxrule. An interactive version of this figure is available at https://blobtoolkit.genomehubs.org/view/ilAriArta2.1/dataset/CALLYF01/cumulative.

**Figure 5.**
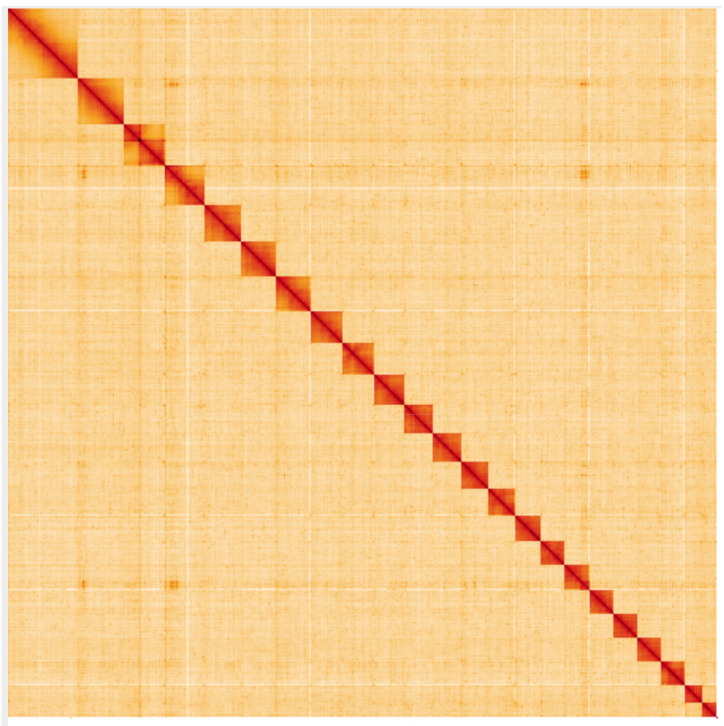
Genome assembly of *Aricia artaxerxes*, ilAriArta2.1: Hi-C contact map. Hi-C contact map of the ilAriArta2.1 assembly, visualised using HiGlass. Chromosomes are shown in order of size from left to right and top to bottom. An interactive version of this figure may be viewed at https://genome-note-higlass.tol.sanger.ac.uk/l/?d=f-4SVoX8TnGQSWWhVc_1WQ.

**Table 2.** Chromosomal pseudomolecules in the genome assembly of *Aricia artaxerxes*, ilAriArta2.

INSDC accession	Chromosome	Size (Mb)	GC%
OW569288.1	1	30.22	37.1
OW569289.1	2	26.28	37.2
OW569290.1	3	25.5	36.9
OW569291.1	4	23.91	36.9
OW569292.1	5	22.53	36.6
OW569293.1	6	22.04	36.9
OW569294.1	7	20.87	36.8
OW569295.1	8	20.11	37.1
OW569296.1	9	19.48	36.8
OW569297.1	10	18.62	36.5
OW569298.1	11	18.4	37.1
OW569299.1	12	17.45	37.1
OW569300.1	13	16.95	37.1
OW569301.1	14	16.61	36.5
OW569302.1	15	15.86	36.9
OW569303.1	16	15.84	37.1
OW569304.1	17	15.65	37
OW569305.1	18	15.62	36.8
OW569306.1	19	15.17	37.4
OW569307.1	20	14.94	37.3
OW569308.1	21	11.45	37.8
OW569309.1	22	9.5	37.8
OW569310.1	Z	45.45	36.2
OW569311.1	MT	0.02	17.5

While not fully phased, the assembly deposited is of one haplotype. Contigs corresponding to the second haplotype have also been deposited.

## Genome annotation report

The GCA_937612035.1 genome assembly was annotated using the Ensembl rapid annotation pipeline ( Table 1; https://rapid.ensembl.org/Aricia_artaxerxes_GCA_937612035.1/Info/Index). The resulting annotation includes 24,684 transcribed mRNAs from 12,597 protein-coding and 2,225 non-coding genes.

## Methods

### Sample acquisition and nucleic acid extraction

A male *A. artaxerxes* (ilAriArta2) was collected from Arthur's Seat, Edinburgh, Scotland (latitude 55.94, longitude –3.16) by Sam Ebdon (University of Edinburgh) and identified by Konrad Lohse (University of Edinburgh) based on wing morphology. The sample was taken from the meadow using hand netting and preserved by freezing at –80°C from live.

DNA was extracted at the Tree of Life laboratory, Wellcome Sanger Institute. The ilAriArta2 sample was weighed and dissected on dry ice with tissue set aside for Hi-C sequencing. Whole body tissue was cryogenically disrupted to a fine powder using a Covaris cryoPREP Automated Dry Pulveriser, receiving multiple impacts. High molecular weight (HMW) DNA was extracted using the Qiagen MagAttract HMW DNA extraction kit. Low molecular weight DNA was removed from a 20 ng aliquot of extracted DNA using 0.8X AMpure XP purification kit. HMW DNA was sheared into an average fragment size of 12–20 kb in a Megaruptor 3 system with speed setting 30. Sheared DNA was purified by solid-phase reversible immobilisation using AMPure PB beads with a 1.8X ratio of beads to sample to remove the shorter fragments and concentrate the DNA sample. The concentration of the sheared and purified DNA was assessed using a Nanodrop spectrophotometer and Qubit Fluorometer and Qubit dsDNA High Sensitivity Assay kit. Fragment size distribution was evaluated by running the sample on the FemtoPulse system.

### Sequencing

A Pacific Biosciences HiFi circular consensus DNA sequencing library was constructed according to the manufacturers’ instructions. DNA sequencing was performed by the Scientific Operations core at the WSI on Pacific Biosciences SEQUEL II (HiFi) instrument. Hi-C data were also generated from whole body tissue ilAriArta2 using the Arima v2 kit and sequenced on the Illumina NovaSeq 6000 instrument.

### Genome assembly

Assembly was carried out with Hifiasm ( Cheng *et al.*, 2021) and haplotypic duplication was identified and removed with purge_dups ( Guan *et al.*, 2020). The assembly was scaffolded with Hi-C data ( Rao *et al.*, 2014) using YaHS ( Zhou *et al.*, 2022). The assembly was checked for contamination and corrected using the gEVAL system ( Chow *et al.*, 2016) as described previously ( Howe *et al.*, 2021). Manual curation ( Howe *et al.*, 2021) was performed using gEVAL, HiGlass ( Kerpedjiev *et al.*, 2018) and Pretext ( Harry, 2022). The mitochondrial genome was assembled using MitoHiFi ( Uliano-Silva *et al.*, 2021), which performed annotation using MitoFinder ( Allio *et al.*, 2020). The genome was analysed and BUSCO scores were generated within the BlobToolKit environment ( Challis *et al.*, 2020). Table 3 contains a list of all software tool versions used, where appropriate.

**Table 3.** Software tools and versions used.

Software tool	Version	Source
BlobToolKit	3.2.9	Challis *et al.*, 2020
Hifiasm	version 0.16.1-r375	Cheng *et al.*, 2021
gEVAL	N/A	Chow *et al.*, 2016
HIGlass	1.11.6	Kerpedjiev *et al.*, 2018
PretextView	0.2	Harry, 2022
purge_dups	1.2.3	Guan *et al.*, 2020
MitoHiFi	2.x	Uliano-Silva *et al.*, 2021
YaHS	yahs-1.1.91eebc2	Zhou *et al.*, 2022

### Genome annotation

The Ensembl gene annotation system ( Aken *et al.*, 2016) was used to generate annotation for the *A. artaxerxes* assembly (GCA_937612035.1). Annotation was created primarily through alignment of transcriptomic data to the genome, with gap filling via protein to-genome alignments of a select set of proteins from UniProt ( UniProt Consortium, 2019).

### Ethics/compliance issues

The materials that have contributed to this genome note have been supplied by a Darwin Tree of Life Partner. The submission of materials by a Darwin Tree of Life Partner is subject to the Darwin Tree of Life Project Sampling Code of Practice. By agreeing with and signing up to the Sampling Code of Practice, the Darwin Tree of Life Partner agrees they will meet the legal and ethical requirements and standards set out within this document in respect of all samples acquired for, and supplied to, the Darwin Tree of Life Project. Each transfer of samples is further undertaken according to a Research Collaboration Agreement or Material Transfer Agreement entered into by the Darwin Tree of Life Partner, Genome Research Limited (operating as the Wellcome Sanger Institute), and in some circumstances other Darwin Tree of Life collaborators.

## Data Availability

European Nucleotide Archive: *Aricia artaxerxes* (northern brown argus). Accession number PRJEB42114, https://identifiers.org/ena.embl:PRJEB42114 ( Wellcome Sanger Institute, 2022). The genome sequence is released openly for reuse. The *Aricia artaxerxes* genome sequencing initiative is part of the Darwin Tree of Life (DToL) project. All raw sequence data and the assembly have been deposited in INSDC databases. Raw data and assembly accession identifiers are reported in Table 1.

## Author information

Members of the Wellcome Sanger Institute Tree of Life programme are listed here: https://doi.org/10.5281/zenodo.4783585.

Members of Wellcome Sanger Institute Scientific Operations: DNA Pipelines collective are listed here: https://doi.org/10.5281/zenodo.4790455.

Members of the Tree of Life Core Informatics collective are listed here: https://doi.org/10.5281/zenodo.5013541.

Members of the Darwin Tree of Life Consortium are listed here: https://doi.org/10.5281/zenodo.4783558.
