# Supplementary material for: The genome sequence of the northern brown argus, Aricia artaxerxes (Fabricius, 1793)
Source: Wellcome Open Res. 2022 Dec 23;7:314. [Version 1] doi: 10.12688/wellcomeopenres.18664.1 (PMC10300502; doi:10.12688/wellcomeopenres.18664.1)
Supplement: Peer review history

# Peer review: Reviewer response for version 1

**DOI:** 10.21956/wellcomeopenres.20697.r58061

**Published:** 2023-08-01

**Authors:** Walther Traut

**Affiliations:** 1 Universität zu Lübeck, Lübeck, Germany

The Ariicia artaxerxes genome is a useful addition to the growing number of good Lepidoptera genomes. Together with the already published lycaenid genomes it opens an excellent basis for research on this family with its excessive variation of chromosome numbers. The quality of the genome assembly is good considering the BUSCO scores. When I searched for telomeres, however, only 9 of the 23 chromosomes had blocks of the insect telomere motif (TTAGG)n or its complement (CCTAA)n on both ends and in the correct orientation. This is not different though from other recently sequenced Lepidoptera genomes.

 The article is clearly written and concise.

 I found some minor points the authors may wish to consider:

- They did not mention how they identified the Z chromosome.
- Figure 2 does not show "white scale lines".
- " *chromosomes are coloured by phylum*" (Figure 3) and " *chromosomes assigned to each phylum*" (Figure 4) are rather cryptic remarks even to a reader familiar with Blob Tools. There is only one phylum in the in the lists.

Are sufficient details of methods and materials provided to allow replication by others?

Yes

Is the rationale for creating the dataset(s) clearly described?

Yes

Are the datasets clearly presented in a useable and accessible format?

Partly

Are the protocols appropriate and is the work technically sound?

Yes

Reviewer Expertise:

NA

I confirm that I have read this submission and believe that I have an appropriate level of expertise to confirm that it is of an acceptable scientific standard.

# Peer review: Reviewer response for version 1

**DOI:** 10.21956/wellcomeopenres.20697.r59310

**Published:** 2023-06-27

**Authors:** Mohadeseh S Tahami

**Affiliations:** 1 University of Jyväskylä, Jyväskylä, Central Finland, Finland

This work is the genome sequencing of one individual of *Aricia artaxerxes*, wild caught from Scotland. The authors present a biological and taxonomic view of the species with its two subspecies; *artaxerxes *and *salmacis. *Later on, they mention that they have used the spotted winged sample for sequencing which makes it the subspecies *Aricia artaxerxes artaxerxes.*

 The species is vulnerable on GB Red List and there is a concern about its demographic and phenological status due to the climate change. 

 All in all, it is a good work, the species background and rationale of the work is well explained. However, since the main outcome of the research is the new genome assembly, more detailed description should be given in the method of assembling and annotation.

 For example, it should be explained if the raw reads were quality checked and trimmed and what was the parameters used for trimming?

 “ *The assembly was scaffolded with Hi-C data (Rao et al., 2014) using YaHS (Zhou et al., 2022).*” Is a little vague to me, needs more clear explanation; was is *de novo* scaffolding? Or did they use a reference-based chromosome assembly?

 It is not clear which sequencing method is to generate genomic data and which is for transcriptomic data?

 The work would be better highlighted to mention that this is the first genome sequencing of this species? if not, better to be compared with other genome assemblies available.

Are sufficient details of methods and materials provided to allow replication by others?

Partly

Is the rationale for creating the dataset(s) clearly described?

Yes

Are the datasets clearly presented in a useable and accessible format?

Yes

Are the protocols appropriate and is the work technically sound?

Yes

Reviewer Expertise:

NGS, phylogeny, genome assembly, taxonomy

I confirm that I have read this submission and believe that I have an appropriate level of expertise to confirm that it is of an acceptable scientific standard, however I have significant reservations, as outlined above.

# Peer review: Reviewer response for version 1

**DOI:** 10.21956/wellcomeopenres.20697.r61102

**Published:** 2023-06-27

**Authors:** Elena A Pazhenkova

**Affiliations:** 1 University of Ljubljana, Ljubljana, Slovenia

The article presents a comprehensive genome assembly of the northern brown argus butterfly, Aricia artaxerxes (Lepidoptera, Lycaenidae). The authors have successfully constructed a high-quality assembly by utilizing data from PacBio long reads and Hi-C data. The resulting genome sequence spans 458 megabases, with an impressive 99.99% of the assembly scaffolded into 23 chromosomal pseudomolecules, including the Z sex chromosome. 

 Gene annotation using Ensembl has identified a total of 12,688 protein coding genes, providing valuable insights into the genetic makeup of the northern brown argus butterfly. The inclusion of taxonomic information, habitat description, and morphological variation across its range enhances the context of the study. The article also highlights the ecological significance and conservation status of the species, emphasizing its vulnerability in the British Isles due to habitat loss, climate change, and potential hybridization with the expanding A. agestis species.

 Overall, this article provides a valuable resource for researchers interested in studying the response of the northern brown argus butterfly biology. The genome assembly presented in this study serves as a solid foundation for further investigations into the species' adaptation and population genomics. The comprehensive information provided, coupled with the attention to detail in assembly refinement, makes this article an important contribution to the field of biodiversity research and genomics.

Are sufficient details of methods and materials provided to allow replication by others?

Yes

Is the rationale for creating the dataset(s) clearly described?

Yes

Are the datasets clearly presented in a useable and accessible format?

Yes

Are the protocols appropriate and is the work technically sound?

Yes

Reviewer Expertise:

Bioinformatics, entomology, ecology

I confirm that I have read this submission and believe that I have an appropriate level of expertise to confirm that it is of an acceptable scientific standard.

# Peer review: Reviewer response for version 1

**DOI:** 10.21956/wellcomeopenres.20697.r59312

**Published:** 2023-06-12

**Authors:** Laurence Despres

**Affiliations:** 1 Univ. Grenoble-Alpes, Grenoble, France

In this article, the authors report on the  chromosome-level genome assembly of the northern brown argus *Aricia artaxerxes* (Lycaenidae), an habitat specialist Palearctic butterfly. The assembly appears to be of good quality using appropriate methods (HiFi long reads, 28-fold coverage, Hi-C data to confirm scaffolding, manual curation of the final assembly) and following the high standard of the Darwin Tree of Life Project. I have only minor comments.

- The final assembly includes 24 scaffolds: 23 chromosomes, including the sexual Z chromosome. Is this chromosome number expected from karyotype data? There is a reference – Federley 1938, in German- but this could be more straight forwardly stated. What is the last scaffold? Is it the mitochondrial DNA? It cannot be the W chromosome as the sequenced specimen is a male.
- The quality of the assembly was further assessed by the proportion of complete BUSCO genes recovered, which is high (>96% of Lepidoptera database BUSCO genes). A full set of BUSCO scores is made available and I was surprised to see that some bacterial or archaea complete BUSCO genes were recovered, although it is stated that the raw reads were cleaned from contamination before analysis. How comes? Does it mean these bacterial/archaea genes are embedded into the butterfly chromosomes? Is it something found in other butterfly genomes?
- For the annotation of the genome, it is mentioned that transcriptomic data were used together with automated annotation but nothing is indicated about the tissue/butterfly stage used for RNAseq.

This high quality chromosome level reference genome of *A. artaxerxes* will be of use to researchers investigating how habitat specialist butterflies respond to climate change.

Are sufficient details of methods and materials provided to allow replication by others?

Yes

Is the rationale for creating the dataset(s) clearly described?

Yes

Are the datasets clearly presented in a useable and accessible format?

Yes

Are the protocols appropriate and is the work technically sound?

Yes

Reviewer Expertise:

NA

I confirm that I have read this submission and believe that I have an appropriate level of expertise to confirm that it is of an acceptable scientific standard.
